# Penile transplantation: A long way to routine clinical practice

**DOI:** 10.12669/pjms.332.11928

**Published:** 2017

**Authors:** Jinhong Li, Feng Qin, Ping Han, Jiuhong Yuan

**Affiliations:** 1 Jinhong Li, MD. Andrology Laboratory/Dept. of Urology, Andrology Laboratory, West China Hospital, Sichuan University, Chengdu, 610000, China; 2 Feng Qin, MD. Andrology Laboratory, Andrology Laboratory, West China Hospital, Sichuan University, Chengdu, 610000, China; 3 Ping Han, MD. Dept. of Urology, Andrology Laboratory, West China Hospital, Sichuan University, Chengdu, 610000, China; 4 Jiuhong Yuan, MD. Andrology Laboratory/Dept. of Urology, Andrology Laboratory, West China Hospital, Sichuan University, Chengdu, 610000, China

**Keywords:** Erectile function, Ethics, Penis, Transplantation

## Abstract

Organ transplantation is an ideal treatment for certain late-stage diseases. With the report of a penile transplantation in Tygerberg Hospital, South Africa, this topic has, once again, aroused exploration in the field. At present, two penile transplantation operations have been performed and gained some positive results. Patients can gain void standing, erectile function and better cosmetic appearance. However, debates and potential risks still exist. Here, we briefly review the progress of studies in this filed and discusses the potential risks and debates in penile transplantation.

## INTRODUCTION

Organ transplantation has been widely used for several end-stage diseases and also benefitted numerous patients.^1^,^2^ With the report of a penile transplantation in Tygerberg Hospital, South Africa, this topic has, once again, aroused exploration in the field.^3^ A penile transplantation is another treatment choice for penile defects, although other methods can be considered, such as penile reconstruction and replantation.^4^-^6^ Conventional penile reconstruction requires multiple operations and implantation of an erectile device, and it may also often result in unsatisfactory cosmetic outcomes.^7^ Long ischemia time and reservation of amputated parts often limit the success of penile replantation.^8^ Based on the above several aspects, penile transplantation may be an ideal method for penile defects.

The first reported penile transplantation was performed in Guangzhou General Hospital of Guangzhou Military Command, China, but it was removed for psychological reasons.^9^ Recently, the so-called first successful penile transplantation was performed in South Africa^3^ Although penile transplantations have been attempted and certain benefits have been obtained, it remains a controversial and challenging issue in medicine.

## RECIPIENTS AND DONORS

The first issue that should be resolved is who needs the operation and who would be the potential donor. Patients with penile carcinoma might be ideal possible patients who require transplantation. Patients who undergo full or nearly total penile amputation often do not resume sexual intercourse, which might be caused by their feeling of shame in having a short penis size.^10^,^11^ Although reconstructive surgery is well established for patients with penectomy, an esthetically perfect penis has not been obtained.^12^ Reconstructive surgery can preserve the ability to void standing and achieve partial sexual function.^13^ However, the poor cosmetic appearance of the penis and/or small penis might affect their psychosexual life. To some extent, penile transplantation can offer patients a penis with a perfect cosmetic appearance. Patients with penile defects caused by trauma may also need penile transplantation. Although penile reconstruction, penile replantation, and penile lengthening are other available options for penile defects, the following limitations should be considered:

- No good substitute is available for erectile tissue in reconstructive surgery
- No excellent cosmetic result can be achieved in most cases
- Multiple operations are needed
- Limited ischemic time and well-preserved amputated penis are required
- Occurrence of nerve/vascular damage.^6^,^12^,^14^

In some countries, many young men lose their penises because of serious complications of traditional circumcision.^15^ Therefore; penis deficiencies caused by surgery might be an important resource for penile transplantation in this area. In addition, patients with surgery-induced severe erectile dysfunction, female to male transsexual or patients with penile inadequacy caused by various reasons can also be considered alternative candidates for penile transplantation.

The source of allograft is a major problem in all transplantation surgery. Similarly, penis allograft is also a challenge because of the specificity of this organ. Brain-dead patients or cadavers might be the main donors in penile transplantation. However, some ethical problems exist. Few patients support organ/tissue donation, particularly involving the privacy of the organ. For their families, a missing penis can mean an imperfection of the body as a man; therefore, penis donation may not be acceptable for them. Individuals who express their willingness to be organ/tissue donors are more likely to be penile allograft donors. Another source of allograft is transgender populations. For male-to-female patients who underwent genital sex reassignment surgery including penectomy, their penis is a precious source for those who need penile transplantation. However, no relevant laws or regulations about the reuse of abandoned organ/tissue in operations are currently in place. In addition, in male-to-female genital sex reassignment surgery, penile skin is important for vaginoplasty.^16^ Skin grafts are needed for these denuded penises, which might increase the difficulty in penile transplantation.

## CONCERNS

Penile transplantation is a new proposition that still faces great challenges. Technically, the development of microsurgery guarantees the connection of nerves/blood. Theoretically, perfect recovery of penile function will be achieved after the operation. To date, to the best of our knowledge, two penile transplantation operations have been performed (Table-I). Superficial/deep dorsal vein, dorsal artery, and dorsal nerve fibers were inosculated in the first penile transplantation operation in China.^9^ In the second reported operation, the surgeons also inosculated three blood vessels, two dorsal nerves, urethra and the corpus cavernosum to ensure sufficient blood flow, restore sensation, achieve urination, and obtain an erection.^3^ However, in the first operation, necrosis occurred at the distal epidermis and no relevant erectile function was reported. Thus, the recovery of sensation and erectile function were not achieved even though the nerve fibers were connected in this operation. Moreover, no official report could be retrieved for the erectile function of the penile transplantation in South Africa. Technically speaking, no problem appears to exist in the penile transplantation operation, but the recovery of erectile function is still worth discussing.

**Table-I:** Summary of Penile Transplantation between 2000 and 2016.

*Patient*	*Date*	*Location*	*Surgical team*	*Donor Details*	*Recipient Details*	*Immunosuppressant*	*Functional Recovery*
Patient 1	2006	Guangzho, China	Guangzhou General Hospital of Guangzhou Military Command, P.R. China	Brain-dead man, age 22 years	Penile defects (traumatic accident), age 44 years	Pre- and intraoperative inducement: Zenapax 50mg, MMF 1000mg, Methylprednisolone 0.5g; Maintenance: MMF 2000mg + Pred 24mg + CsA 420mg	Void standing at day 10, No sexual function was reported (transplanted penis was cut off at day 14)
Patient 2	2014	Cape Town, South Africa	Tygerberg Hospital, Stellenbosch University, South Africa	Not reported	Penis amputee (failed ritual circumcision), age 21 years	Referred to other composite tissue transplants (hand and face transplants)	Full sexual and urinary function

MMF:Mycophenolate mofetil; Pred:Prednisone; CsA:Cyclosporine

The agents of immunosuppression used for penile transplantation may be complicated because of composite tissue allograft. In the first penile transplantation,^9^ pre and intra-operative immunosuppressive induction regimens were administered [50 mg of Zenapax, 1000 mg of mycophenolate mofetil (MMF) at 1.5 h preoperatively, 0.5 g of methylprednisolone intraoperatively, and 0.5 g of methylprednisolone postoperatively on days 1, 2, and 3]. Drugs used in the maintenance treatment were MMF, prednisone, and cyclosporine (2000 mg/day MMF, 24 mg/day prednisone, and 420 mg/day cyclosporine). Transplanted penile shaft (2 weeks postoperatively, amputated because of a psychological problem) showed no rejection under microscopy as reported; however, only the finding in microscopy was not considered for graft acceptance.^16^ Thus, this protocol of immunosuppression, similar to that of kidney transplantation, is controversial. Immunosuppressive agents used in another penile transplantation, according to media statement,^17^ referred to face and hand transplantations, but no detailed information was reported. Penile transplantation is unlike other organ transplantations that, to some extent, are life-saving; however, it is certainly life-changing. According to the reported data, lifelong immunosuppression might result in cancer, infections, metabolic disorders, and even death.^18^ The application of immunosuppressive agents might be a possible factor influencing the quality of life. The side effects brought by immunosuppression, such as diabetes mellitus, may affect erectile function.^19^ Thus; we should weigh the advantages and disadvantages between the allograft protection and the adverse events caused by the postoperative immunosuppressive agents. At present, no guide can be followed for the use of immunosuppressive agents in penile transplantations. The selection of reasonable immunosuppressive drugs remains a great challenge and needs further exploration. In general, immunosuppressants should refer to some composite tissue transplantations, such as face/hand transplantations.

Psychological issues not only affect the recipients but also their sexual partners. The penis is unlike other allografts, which are classified as invisible organs. Penile transplantation recipients can see and even touch this allograft. For recipients, seeing the allograft might often remind them of the penis donors. The recipients may even have difficulty accepting the graft as their own body subconsciously. According to data recently reported by Gross, 22 recipients of the transplantations at the University of Minnesota (since 1970 to 2006) committed suicide.^20^ Although the specific reason for suicide is unclear, potential psychological factors should not be ignored. The penis of the recipient in the first penile transplantation was cut off because of psychological problems of the patient and his sexual partner.^9^ Thus, pre and post operative psychological guidance should be effectively implemented. For sexual partners of recipients, accepting another penis other than their husband is difficult because of the influence of traditional ideology. Both recipients and their sexual partners require some time to adjust to the penile transplantation and integrate it into their own lives.

## CONTROVERSIES AND FUTURE DIRECTIONS

Although two penile transplantation operations have been performed and gained some positive results, this procedure is still a topic of much debate and holds potential risks. In addition, many questions remain unanswered in this newly emerging field.

The success of the operation is closely related to the selection of recipients. As aforementioned, psychological issues is critical in penile transplantation. Therefore, an ideal candidate is one who has finished pre-operative assessment with positive results. Patients with psychological problems should not be considered for this transplantation. Furthermore, the application of preventive postoperative mental counseling is recommended. Similarly, psychological counseling for sexual partners of recipients is also advised, which can prevent some potential psychological burden from the wife’s side.

A penile transplantation involves severe ethical issues among the medical community. First, the penis is a relatively private organ in the human body. Donors have difficulty signing penile transplantation on the voluntary organ donation instrument, or they are more willing to donate some other organ, such as the kidney, liver, and cornea. Their families also have difficulty accepting the loss of loved ones and loss of their loved ones’ penis. From the traditional perspective of some countries, the penis is a sign of masculinity, and preserving the integrity of the body is considered a sign of respect for the dead by the loved ones. Second, whether the penile transplantation is the best choice for individuals is difficult to assess. The benefits of surgery and its associated complications should be balanced. Recipients can gain void standing, relatively better cosmetic appearance, and erectile function. However, this newly innovative therapy might bring potential disadvantages caused by surgery itself and also by life-long post-operative medicine. For patients, deciding which method to choose is difficult. Thus, patient selection mainly relies on surgeons. Choosing the right patient might determine the success or failure of the operation. Given that the aim of penile transplantation includes the recovery of erectile function, preoperative erectile function should be evaluated. Third, whether this procedure is mature enough to be applied to human beings is unclear and the informed consent process is very complex. The recipients should be fully informed that this surgery presently belongs to an experimental attempt, and many unknown risks exist. The patients should also be informed about alternative therapies, except for penile transplantation.

The aims of penile transplantation for recipients are void standing, cosmetic appearance, and erectile function. In the first attempt at a penile transplantation, the penis was cut off at 14 days postoperatively;^9^ therefore, no erectile function information was collected. Although full sexual function was reported for the second penile transplantation, no long-term data could be obtained to detect erectile function because the effects of the application of long-term immunosuppressive agents on sexual function remain unclear.^3^ In addition, whether the inosculated nerves will restore erectile function is inconclusive in penile transplantations. What will happen if the recipients do not recover their erectile function but achieve void standing and a good cosmetic appearance? Will this case be classified as a successful surgery or not? Will another penis be transplanted or will other remedial measures be considered? All of these questions are difficult to answer.

At present, only two penile transplantations were reported but showed initial success. However, uncertainty of long-term erectile function, immunosuppression-related concerns, ethical issues, and even financial burden has limited the development of penile transplantations. Penile transplantation is still in the exploratory stage, and the operation will be performed only after strict preoperative evaluation, patient selection, full informed consent, and approval of the ethics committee. Only after all the above concerns are solved can penile transplantation be applied in routine practice.
